# Dual Graded Lattice Structures: Generation Framework and Mechanical Properties Characterization

**DOI:** 10.3390/polym13091528

**Published:** 2021-05-10

**Authors:** Khaled G. Mostafa, Guilherme A. Momesso, Xiuhui Li, David S. Nobes, Ahmed J. Qureshi

**Affiliations:** 1 Additive Design and Manufacturing Systems (ADaMS) Lab, Department of Mechanical Engineering, University of Alberta, Edmonton, AB T6G 1H9, Canada; kmostafa@ualberta.ca (K.G.M.); xiuhui1@ualberta.ca (X.L.); 2 Independent Researcher, São Paulo 05508-060, Brazil; guilherme.momesso@alumni.usp.br; 3 Department of Mechanical Engineering, University of Alberta, Edmonton, AB T6G 1H9, Canada; dnobes@ualberta.ca

**Keywords:** lattice, grading, infill, relative density, unit cell, size, porosity, triply periodic minimal surface, absorbed energy, digital image correlation, stereolithography, 3D printing

## Abstract

Additive manufacturing (AM) enables the production of complex structured parts with tailored properties. Instead of manufacturing parts as fully solid, they can be infilled with lattice structures to optimize mechanical, thermal, and other functional properties. A lattice structure is formed by the repetition of a particular unit cell based on a defined pattern. The unit cell’s geometry, relative density, and size dictate the lattice structure’s properties. Where certain domains of the part require denser infill compared to other domains, the functionally graded lattice structure allows for further part optimization. This manuscript consists of two main sections. In the first section, we discussed the dual graded lattice structure (DGLS) generation framework. This framework can grade both the size and the relative density or porosity of standard and custom unit cells simultaneously as a function of the structure spatial coordinates. Popular benchmark parts from different fields were used to test the framework’s efficiency against different unit cell types and grading equations. In the second part, we investigated the effect of lattice structure dual grading on mechanical properties. It was found that combining both relative density and size grading fine-tunes the compressive strength, modulus of elasticity, absorbed energy, and fracture behavior of the lattice structure.

## 1. Introduction

Additive manufacturing (AM) has triggered a paradigm shift towards parts and assemblies design methodologies, materials development and utilization, and supporting the parts’ additional functionalization [1,2]. With the increasing number of successful case studies in biomedical, aerospace, and automotive sectors [3], AM is disrupting more industrial sectors from confectionaries [4] and shoemaking [5] to oil and gas [6] and constructions [7]. Several polymer and metal additive manufacturing processes can manufacture parts with significant geometrical and dimensional accuracy [8,9,10,11,12]. AM unleashes the manufactured parts’ geometric freedom, enabling designers to fully utilize topology optimization and complex lattice structures to design functional parts while reducing the part’s weight or tailoring its thermo-mechanical properties [13,14,15,16].

**Figure 1:**
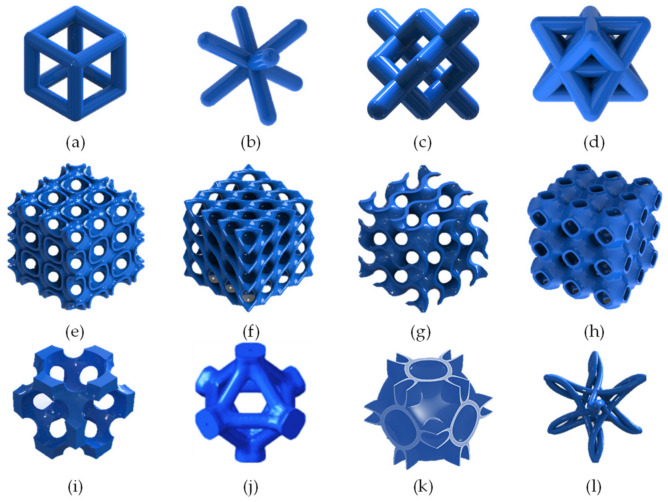
Different types and shapes of unit cells: (**a**) simple cube, (**b**) back-centered cubic BCC, (**c**) diamond, (**d**) octet-truss, (**e**) IWP Schoen, (**f**) Schwartz diamond, (**g**) gyroid, (**h**) Schwartz primitive, (**i**,**j**) topology-optimized face-centered cubic cells, (**k**) hybrid (Neovius + Schwartz P), and (**l**) custom artistically designed unit cell.

Lattice structures are a type of cellular material characterized by open porosity and can be arranged in a stochastic or non-stochastic unit cell order [17]. Non-stochastic lattice structures are constructed by the periodic repetition of an elementary unit called a unit cell within a given domain along the principal axes [18]. A unit cell’s geometry is classified into four major groups: strut-based, triply periodic minimal surfaces (TPMS), topology-optimized (TO), nature-inspired, and custom-designed. Several strut-based unit cells are based on the geometry of the Archimedean solid, for example, the simple cube, body center cubic (BCC), diamond, and octet truss, as shown in Figure 1a–d. The TPMS are implicit surfaces with a zero mean curvature [19], for example, Schoen IWP, Schwartz D, gyroid, and Schwartz P, as shown in Figure 1e–h. Topology-optimized unit cells (TO) are generated using a finite element method-based optimization to minimize or maximize a specific mechanical property [20]. Several TO units developed recently; some of the famous designs are face-centered cubic (FCC) and reverse FCC unit cell [21] as shown in Figure 1j. Some unit cells are inspired by structures found in nature, such as honeycombs, plants, bubbles [22], and human and animal bones [23,24,25]. The last group is the custom-designed lattices, such as hybrid unit cells that achieve cross-performance of two different unit cells [26], as shown in Figure 1k. Custom cells also include auxetic unit cells, which achieve negative Poisson’s ratio [27] or negative thermal expansion coefficient [28]. There are also aesthetics-designed unit cells [29], as shown in Figure 1l. Lattice structure utilization has enhanced several applications, such as improving the oxygen permeability, mechanical properties, and biodegradability of biomedical implants [30,31,32]. It was also used to improve antennas and acoustic devices’ performance while maintaining a lightweight and compact design [33,34].

The lattice structures’ mechanical properties can be predicted as a function of their unit cell geometry, relative density, size, porosity, shell or strut dimensions, and spatial orientation [35,36,37,38]. A minor modification in any unit cell parameters significantly affects the whole lattice structure’s mechanical behavior [39]. Most lattice structures are stretching-dominant structures while undergoing tensile or compressive stresses and provide a stiff structure. However, some lattice structures are bending-dominant structures with higher energy absorption capacity [40,41,42]. TPMS unit cells have an above-average surface area with low stress generated during static loadings as well as better handling of angular loads as a result of the better load distribution compared to strut-based design strut-based lattices [43,44]. Topology-optimized lattices possess higher stiffness at lower relative density than TPMS and strut-based lattices [45]. In general, the unit cell’s relative density is the most significant parameter in determining the overall stiffness and the ultimate tensile strength [43,45]. Unit cell size, however, has a crucial role in determining the failure mechanism of the structure. Smaller cell size improves the low strain structural failure due to a localized fracture [46] and enhances the buckling resistance [47]. As the cell size increases, energy absorption decreases [48]. The stress-strain curve’s compressive plateau value and the strain value at which the plateau occurs are controlled by the unit cell’s relative density and size combined. As the unit cell’s relative density increases and its size decreases, the unit cells’ self-contact occurs and gets closer to densification, which decreases the strain required to reach the plateau and also produces a smoother curve with minimal stress fluctuation [40,43].

Another advantage of additive manufacturing is the functional grading of the lattice structures, materials, and microstructures [17]. Functionally graded structures offer part optimization and property tailoring over the part domain, such as tuning a part’s natural frequency [49] or thermal conductivity [50] while maintaining the part strength. Moderate relative density grading was found to improve the stiffness of the part significantly [51]. Size grading has improved energy absorption in some loading directions and did not affect other directions. Relative density grading was confirmed with another study to be the most significant parameter to control the part stiffness and energy absorption [52,53]. The grading equation was shown to affect the part’s behavior, for example, linear or harmonic grading [54].

Lattice structure generation requires two main processes: unit cell modeling and unit cell propagation. The unit cell is modeled and represented, currently, by three main methods. The unit cell is represented as an implicit function, voxel logical array, or CAD file, usually a surface mesh representation [18,22,55]. Implicit modeling allows the control of the relative density and the size of the unit cell. However, it requires computational power to compute the surface for each point in the part domain. The voxel logical array and the STL file can only scale the cell size without controlling the unit cell relative density.

The unit cell propagation methods are classified into conformal or direct patterning [42,56]. Conformal patterning allows unit cells to conform and orient with respect to the domain boundary and space. In contrast, direct patterning uses the domain’s boundary to trim the uniformly tessellated unit cells all over the domain space. The integrity of the cells in conformal patterning methods is better compared to direct patterning. However, the unit cell’s size, orientation, and aspect ratio are dependent on the domain geometry, which might affect the part’s intended mechanical properties. Direct patterning allows propagating the cells with independent parameters from the part geometry, facilitating the tailoring of the part properties. A solution to the trimmed direct-patterned lattice structure integrity is to use a solid or a conformal designed shell on the part boundary [18]. Propagation of implicitly represented unit cells allows for a smoother transition between different density grading, which decreases the stress concentration [57] compared to the voxel and the CAD representation method, which requires extra processing steps to fill the gaps [58].

The implicit grading of the cell type, relative density, and size of the TPMS can be performed using MS lattice software based on a user-defined grading function; however, it does not achieve a dual graded lattice structure [52,55]. The implicit unit cell generation method can provide relative density grading while manipulating unit cell distortion (size, skewness, and rotation) to follow stress contours generated in different bodies [59]. Several available commercial software provides lattice structure modeling and topology optimization, for example, Netfabb™, 2020, Autodesk, CA, USA [60], Inspire™, 2020, Altair™, MI, USA [61], ANSYS, 2020, ANSYS™, PA, USA [62], 3-Matic™, 2020, Materialise, Leuven, Belgium [63], ParaMatters™, 4.0, ParaMatters™, CA, USA [64], and n-Topology™, 2.0, n-Topology, NY, USA [65].

One of the research gaps found in the literature is that the effect of applying two simultaneously varying properties of the unit cells within the lattice structure on mechanical properties is not investigated. This type of grading is called dual grading. The grading function and grading direction have not been sufficiently studied, and the grading direction, whether relative density or size grading, is unidirectional and symmetric around one axis only. Additionally, the effect of the unit cell’s aspect ratio grading on the mechanical properties was not sufficiently studied experimentally. This paper presents a framework that allows for dual graded lattice structure generation. The user can simultaneously achieve both size and relative density grading while propagating various custom unit cell types within the different part’s domains. The dual grading designs allow harnessing the benefits of both types of grading. With the continuous development of new unit cell geometries, there is a need for a generic framework that can adopt the new unit cell quickly. The currently available commercial and open-source software limits the unit cell types used to generate a lattice structure to pre-defined unit cells. The dual graded lattice structure generation framework (DGLS) allows the users to input any custom unit cell. The DGLS manipulates the size and the relative density grading independently based on a user’s custom input mathematical functions in terms of the unit cell’s spatial position.

The first part of the paper describes and discusses the algorithm and the software developed to achieve a dual graded lattice structure using several case studies. The second part of this paper experimentally investigates the dual grading, grading functions, and grading direction effects on the tuning of mechanical properties. Contrary to the unilateral and non-symmetrical graded test specimen found in literature, this paper investigates bilateral and fully symmetrical grading around the test specimens’ geometrical center. The lattice specimens’ deformation was monitored and analyzed using digital image correlation (DIC), and the fracture shape was captured.

## 2. Dual Grading Generation Algorithm

**Figure 2:**
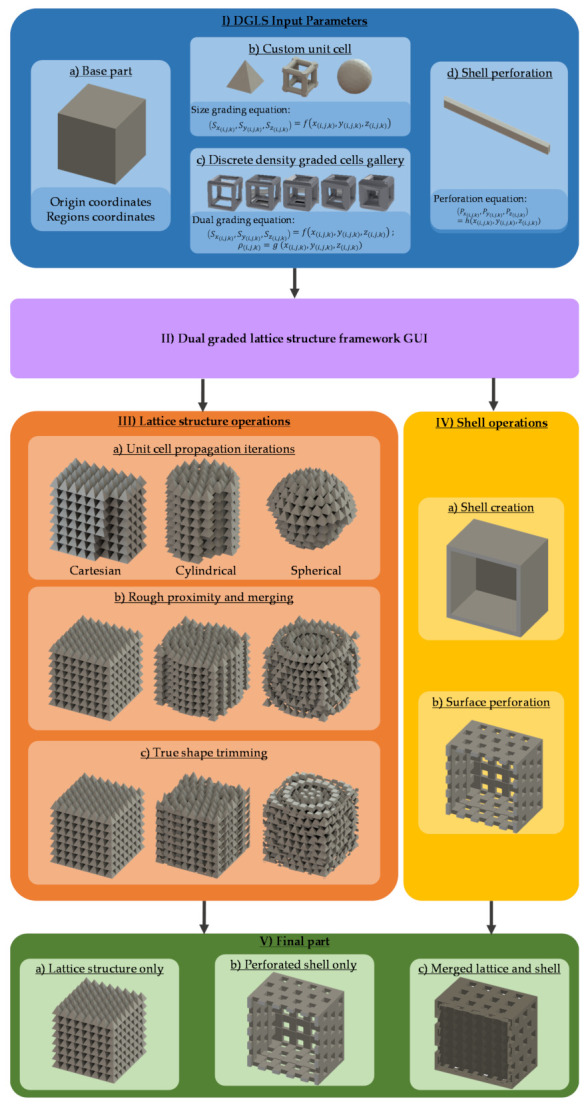
Dual grading generation framework: (**I**) Input files and parameters, (**II**) graphical user interface, (**III**) lattice structure operations, (**IV**) shell operations, and (**V**) final part.

The dual graded lattice structure framework (DGLS) is developed to allow the user to vary/grade two properties of the unit cells simultaneously while generating the lattice structure. The DGLS algorithm consists of five main processes, as shown in Figure 2:

1. Inputting of computer graphics parameters (Process I)
2. Inputting of DGLS GUI parameters (Process II)
3. Performing of lattice structure (Process III)
4. Performing of shell operations (Process IV)
5. Exporting final part (Process V)

Each process has different options or sub-processes that determine the final output geometry. Each process requires specific user inputs, either numerical equations, constants, or STL files. After the user provides the required inputs, the lattice structure and shell operations start. The processing time varies based on the resolution of the STL files, the size of the unit cells compared to the base part, and if the shell operations are required or not. The user can export the lattice structure, perforated shell, or the merged shell and lattice structure as the final part STL file.

### 2.1. Development Environment

The DGLS framework was developed in a C++ environment with a basic graphical user interface (GUI) built in the Qt framework. This environment can take advantage of the powerful graphics and mathematical libraries available in open-source formats. Some examples include the geometry processing library (LIBIGL) [66], computational geometry algorithms library (CGAL) [67], OpenGL Mathematics (GLM) [68], Cork Boolean library (CORK) [69], the matrix and linear algebra library EIGEN [70], and the mathematics expression parser and compiler TINYEXPR [71].

### 2.2. DGLS Input Parameters

As shown in Figure 2, the first input parameter is the base part (I-a), a binary STL file, to be infilled with the lattice structure. After that, the user either accepts the current part origin point, which by default is set to the part geometric center or assigns a new origin relative to the original one. Afterwards, the user has to define the different part domains/regions. Each domain can have its unique unit cell geometry, grading equations, coordinate system type, and sub-origin point. The part can be divided into an unlimited number of domains. For each domain, the user has to input the appropriate STL file for the lattice structure’s unit cell. The user can then configure the domain boundary shape and size.

In the case of a size graded or non-graded lattice structure, one STL file for the unit cell (I-b) is required per each part domain. However, for the relative density, porosity, or dual graded lattice structure, a discretized gallery (I-c) of at least two different relative density unit cells needs to be input along with their relative density numerical values. The unit cell size is manipulated independently in the unit cell’s three main axes: the $S_{x_{\left(i,j,k\right)}}$, $S_{y_{\left(i,j,k\right)}}$, and $S_{z_{\left(i,j,k\right)}}$, where $\left(i,j,k\right)$ is the position index of the unit cell in the lattice structure. This design feature allows the aspect ratio of the unit cells to be controlled throughout the lattice structure. The user has to define three equations to describe the size as a function of the unit cell’s three-dimensional position relative to the origin of the corresponding part’s domain.

For relative density grading, the user has to provide one equation describing the relative density grading, $\rho_{\left(i,j,k\right)}$, as a function of the unit cell’s three-dimensional spatial position relative to the domain’s origin. For dual grading, both the size and relative density equations are required. The spatial position used in defining the grading equations can follow either a Cartesian, a cylindrical, or a spherical coordinate system. The coordinate system dictates the unit cells’ propagation directions. Equations can contain a wide range of mathematical functions from linear and polynomial to periodic and logarithmic functions.

The shell operations require the user to input the shell thickness, perforation pattern tool STL file (I-d), and a user-defined equation to describe the perforations’ in-plane position as a function of in-plane coordinates. The user has to set an overlap value percentage, which describes the amount of the overlapping distance between any two cells in any axis as a function of the current unit cell size. The overlap percentage is evaluated for each propagation iteration. The overlap is set to ensure the lattice structure’s integrity.

### 2.3. Lattice Structure Operations

The lattice structure operations (III) consist of three sub-processes: the unit cell propagation (III-a), the rough proximity and merging (III-b), and the true shape trimming (III-C), as shown in Figure 2. If the part is portioned in several domains, the cell propagation process is performed independently for each part domain using the domain’s origin. Otherwise, it propagates through the whole part and uses the whole-body origin. The propagation process (III-a) has three different iterators; each one is dedicated to a particular coordinate system. The propagation direction follows the coordinate system that was chosen for the domain or the whole body. All iterators set the first cell at the origin point (0,0,0) regardless of the coordinate system.

The first iterator is dedicated to Cartesian coordinates. The Cartesian iterator reads the input size equations and evaluates the unit cell’s size at the origin, then sets the first unit cell at the domain’s origin. The iterator then evaluates and updates the new *z*-position based on the current unit cell size in the *z*-direction ($S_{z_{\left(i,j,k\right)}}$) at position index of $\left(i,j,k\right)\;$and the overlap value (δ), as represented by (6). Subsequently, it evaluates the new cell three-dimensional size (*S_x_*, *S_y_*, and *S_z_*) at the new evaluated position ($x_{\left(i,j,k+1\right)},y_{\left(i,j,k+1\right)},\;\mathrm{and}\;z_{\left(i,j,k+1\right)})$ and adds the new cell at the newly evaluated *z*-position. After the iterator continues the propagation in the *z*-direction till the unit cells reach the rough proximity boundary of the domain or the part, it sets the *z*-position to zero again. The iterator evaluates the new *y*-axis position based on the current unit cell size in the *y*-direction ($S_{y}{}_{\left(i,j,k\right)}$) and the overlap value$\;\left(\delta \right)$, as shown in (5). Then, the iteration in the *z*-direction is repeated. The iteration in the *y*-direction continues until the unit cells reach the part’s or the domain’s rough proximity boundary. Then, it sets the *y*-position to zero again. Then, the iterator evaluates the new *x*-position based on the current unit cell size in the *x*-direction ($S_{x}{}_{\left(i,j,k\right)}$) and the overlap percentage. Subsequently, the iteration in the *y*-direction is repeated. It is similar to nested loops. The x-direction’s iterations are carried out in the outer loop, the *y*-direction’s iterations in the middle loop, and the *z*-direction’s iterations in the innermost loop.

For the relative density grading or dual grading required by the user, the iterator initially reads the relative density grading equation and the discretized unit cell gallery. Then, the relative density is evaluated in the *z*-loop iterations for each cell. The relative density value is rounded to the nearest available unit cell from the discretized unit cell gallery inputted by the user. The evaluated cell size is applied to the chosen unit cell from the gallery before adding it to the lattice structure. If the part has multiple domains, the *x*, *y*, and *z* iterations are applied to each domain independently in the ascending order in which the domains were defined.

For the size grading, the DGLS framework was developed to have one independent size axis, and the other two size axes can either be set to be the independent or dependent axis. In the case of the Cartesian iterator, the $S_{x_{\left(i,j,k\right)}}$ can be only a function of the *x*-axis location as follows:

(1) $$S_{x_{\left(i,j,k\right)}}=f_{x}\left(x_{\left(i,j,k\right)}\right)$$

The $S_{y_{\left(i,j,k\right)}}$ can also be a function of *y*-axis location only or both *x* and *y*-axis locations, as follows:

(2) $$S_{y_{\left(i,j,k\right)}}=f_{y}\left(x_{\left(i,j,k\right)},y_{\left(i,j,k\right)}\right)$$

The $S_{z_{\left(i,j,k\right)}}$ can either be a function of the *z*-axis location only or all the three axes locations as follow:

(3) $$S_{z_{\left(i,j,k\right)}}=f_{z}\left(x_{\left(i,j,k\right)},y_{\left(i,j,k\right)},z_{\left(i,j,k\right)}\right)$$

The new cell location in each iterator is calculated using the current cell location, the size of the current cell, and the overlap value as follows:

(4) $$x_{\left(i+1,j,k\right)}=x_{\left(i,j,k\right)}+S_{x}{}_{\left(i,j,k\right)}-\delta /2$$

(5) $$y_{\left(i,j+1,k\right)}=y_{\left(i,j,k\right)}+S_{y}{}_{\left(i,j,k\right)}-\delta /2$$

(6) $$z_{\left(i,j,k+1\right)}=z_{\left(i,j,k\right)}+S_{z}{}_{\left(i,j,k\right)}-\delta /2$$

These constraints assure that most of the size grading equations lead to a successful lattice structure where all the unit cell nodes are attaching to the neighbouring unit cells’ corresponding nodes. The axis dependency is further discussed in the case studies section. The relative density grading can be a function of any of the axes or all the axes together.

The cylindrical and spherical iterators are similar to the Cartesian iterator algorithm, in which a nested loops structure was used. The only difference is the axes’ names and the propagation directions. For the cylindrical iterator, the unit cell propagation starts in the *z*-axis direction, then the iterator shifts the polar direction, *θ*. After the *θ* iterations reach 180°, the iterator shifts the *r*-position in the radial direction. Therefore, the outer loop controls the propagation in the radial direction, the middle loop controls the polar direction, and the inner loop controls the *z*-direction. The spherical iterator’s outer loop controls the propagation in the radial direction. The middle loop controls propagation in the polar direction, and the inner loop controls the azimuthal direction, ϕ. The size dependency on the coordinate axes for the cylindrical iterator is described by:

(7) $$(S_{x_{\left(i,j,k\right)}},S_{y_{\left(i,j,k\right)},}\;S_{z_{\left(i,j,k\right)}})=\left(f_{x}\left(r_{\left(i,j,k\right)}\right),f_{y}\left(r_{\left(i,j,k\right)},\theta_{\left(i,j,k\right)}\right),f_{z}\left(r_{\left(i,j,k\right)},\theta_{\left(i,j,k\right)},z_{\left(i,j,k\right)}\right)\right)\;$$

while the size dependency on the coordinate axes for the spherical iterator is described by:

(8) $$(S_{x_{\left(i,j,k\right)}},S_{y_{\left(i,j,k\right)},}\;S_{z_{\left(i,j,k\right)}})=\left(f_{x}\left(r_{\left(i,j,k\right)}\right),f_{y}\left(r_{\left(i,j,k\right)},\theta_{\left(i,j,k\right)}\right),f_{z}\left(r_{\left(i,j,k\right)},\theta_{\left(i,j,k\right)},\phi_{\left(i,j,k\right)}\right)\right)$$

It is worth noting that the DGLS currently does not support the unit cell rotation during the propagation; therefore, the unit cell’s angular orientation is set by the original STL file orientation.

The second sub-process in the lattice structure operations is the merging of unit cells (III-b). This process takes place directly after the unit cell is propagated to fill roughly every domain. The unit cells within each domain are merged using Boolean operations. Two options are available for the domain’s rough-shaped lattice. It can be trimmed with the shape of the domain boundary, or the roughly shaped lattice gets merged with the surrounding untrimmed domains. The second option ensures that the lattice structure is one connected part.

The last process is the final shape trimming of the whole part (III-c). After all the domains are merged, the semi-finished lattice structure is trimmed with the base part’s boundary if no shell is required. Otherwise, the semi-finished lattice structure is trimmed with the offset boundary surface of the shell generated.

### 2.4. Shell Operations and Part Finishing

The user can create a shell out of the base part using the shell operations process (IV), as shown in Figure 2. The shell is created using operation (IV-a) by offsetting the base part’s external surface inwards by a distance equal to the shell thickness, then subtracting the newly formed part from the original base part using Boolean operations. Several issues, such as global loops and the self-intersecting local loops, may arise depending on the part geometry and the shell thickness value. The DGLS framework utilizes the bi-arcs fitting algorithm [72] to detect and solve global or local loops.

The user can choose to perforate the created shell using operation (IV-b), which requires the custom patterning tool STL file (I-d). The patterning tool is replicated over the three principal planes: *x–y*, *x–z*, and *y–z*. A user-defined position function governs each replication position in terms of the in-plane Cartesian coordinates. Afterwards, the pattern replication over each plane is projected on the body and subtracted using Boolean operations. Currently, the DGLS is using a Cartesian coordinates system in the shell perforation. The user can choose to export the resultant final part (V) as a lattice structure without the shell (V-a), a hollow perforated shell without any lattice structure inside (V-b), or a merged lattice structure with a generated shell (V-c). The output part is exported as an STL file.

## 3. Case Studies

Several test scenarios were designed to assess the DGLS framework’s performance. The test scenarios included the generation of a constant size lattice structure, size graded, and dual graded lattice structure. For each scenario, a different unit cell type and geometry are used.

**Figure 3:**
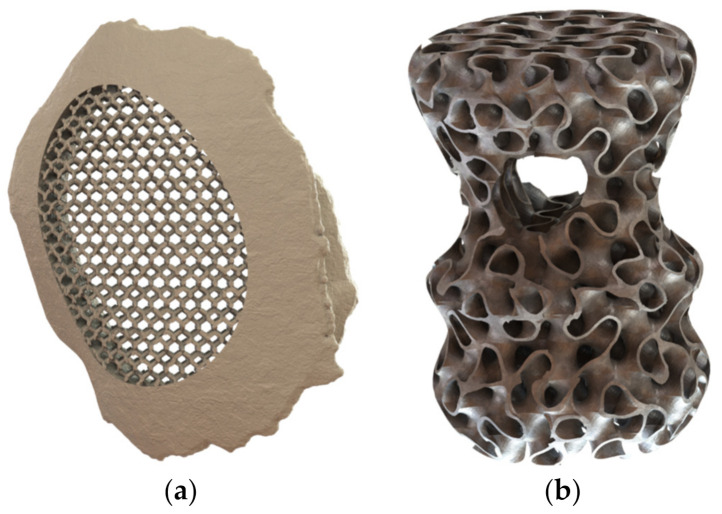
Constant size and relative density generated lattice structure for (**a**) skull implant partially filled with a cubic diamond lattice structure and (**b**) spline-based revolved artifact-filled gyroid.

For the constant lattice structure generation, two scenarios were envisaged. The first one uses a cubic diamond unit cell, which belongs to the strut-based unit cells, to fill an ellipse-shaped domain within a skull implant base part, as shown in Figure 3a. The second scenario uses the gyroid unit cell, which belongs to the TPMS unit cells, to fill a whole artifact base part shaped as a revolved spline with an ellipse cut-out, as shown in Figure 3b. The size equations for both scenarios were set to constant values, and the coordinate system was set to Cartesian. The output parts were digitally examined and qualitatively evaluated to locate any mesh discontinuities. Both parts showed a successfully generated lattice structure.

**Figure 4:**
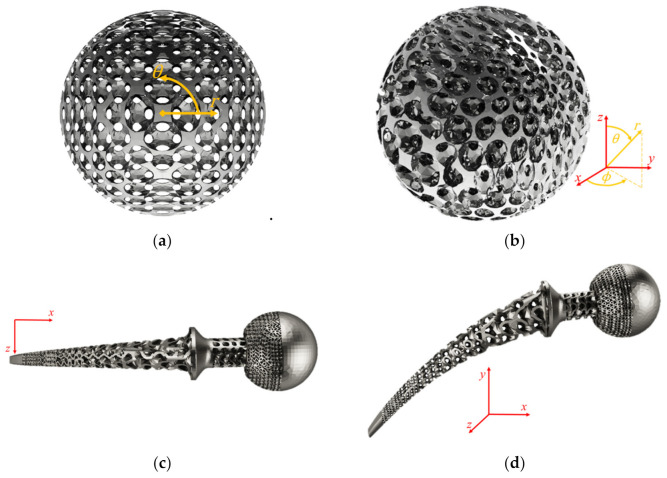
Size graded lattice structures: (**a**) side view and (**b**) isometric view of a sphere filled with TO FCC unit cell and (**c**) top view and (**d**) isometric view of multi-domain hip joint implant partially filled with Schwartz D unit cell.

For size grading, the first testing scenario was to fill a sphere base part with a size-graded topology-optimized FCC unit cell using a spherical coordinates system, as shown in Figure 4a,b. Each of the size grading governing equations depended on one unique coordinate axis only. This design allows for a spatially graded unit cell size with a varying aspect ratio while constraining each unit cell’s nodes to meet all neighbouring unit cells. The origin of the coordinate system was set to be in the center of the sphere. The unit cells’ *S_x_* varied linearly in the radial direction with the largest size value at the center and decreased towards the sphere circumference. Simultaneously, the *S_y_* slightly varied in the polar direction in a sinusoidal behavior as a function of the polar coordinate. The *S_z_* was set to vary sinusoidally in the azimuthal direction. The second scenario was to partially infill a hip joint implant with a size-varying Schwartz D, which belongs to the TPMS unit cells using Cartesian coordinates, as shown in Figure 4c,d. The part was divided into six domains, some of the domains were left solid, and the others were infilled with the Schwartz D unit cell either with size graded or constant unit cells. The three size equations were dependent on the *x*-axis only. This design allows for a fixed aspect ratio while applying the size grading.

**Figure 5:**
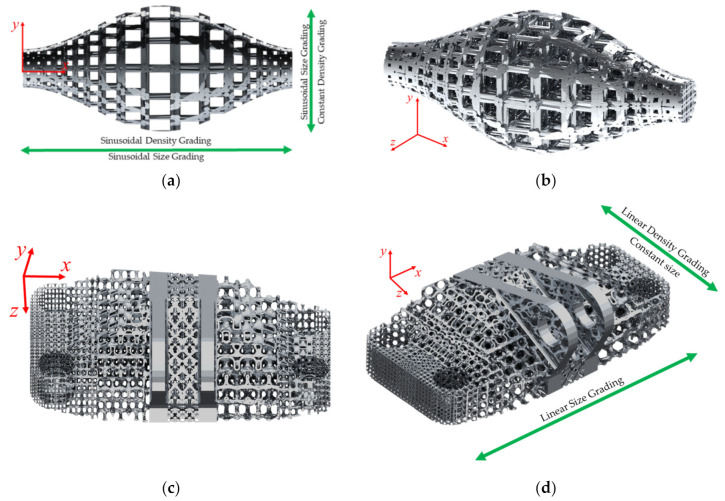
Dual graded lattice structure: (**a**) Front view and (**b**) isometric view of a sinusoidal-based revolved parts filled with a cubic unit cell and (**c**) top-down view and (**d**) isometric view of GE bracket filled with IWP Schoen unit cell.

For the dual grading, two test scenarios were designed to test DGLS’s ability to handle dual grading for strut-based and TPMS unit cells and linear and non-linear grading. The first testing scenario was to infill a sinusoidal revolve shape with a simple cubic unit cell using Cartesian coordinates, as shown in Figure 5a,b. The origin was set to the center of the shape. The discretized relative density graded unit cell gallery used consisted of five different relative densities. The size followed a sinusoidal grading function for the three size equations, with the largest unit cell size in the center of the part and a size decrease towards the part external surface. The *S_x_* was only a function of the *x*-axis, the *S_y_* was interdependent on both the *x* and the *y* axis, and the *S_z_* was interdependent on all three axes. The size interdependency on multiple axes was successful with the framed unit cells, while for the nodal-based unit cell, it produced gaps between the neighbouring nodes. The relative density grading followed a sinusoidal grading function as a function of the *x*-axis only. The densest unit cells were on the edges, and the relative density decreased towards the center of the part.

The second scenario for the dual grading was to fill the well-known GE bracket artifact with a varying porosity IWP Schoen unit cell using a Cartesian coordinate system, as shown in Figure 5c,d. The part was partitioned into five domains. The middle domain consisted of a non-graded octet truss lattice structure; next to it, from both sides, is a complete solid domain and surrounded by the dual-graded IWP Schoen lattice structure. The discrete unit cell gallery consisted of three unit cells with a varying ISO-value ranging from 0.3 to 0.9. Size grading followed a linear function and was varying only along the *x*-axis for the three size equations. It started with a small unit cell size near the part’s edges and increased towards the center. The porosity grading also followed a linear function, and it was varying in the orthogonal direction along the *z*-axis. It started with a smaller pore size at the center of the part, and the pore size increased towards the outer edges. The dual grading algorithm was successful with both the strut-based and the TPMS based unit cells. It was observed that the size of the discretized gallery and the slope of the grading function affect the smoothness of the transition between different relative density cells.

**Figure 6:**
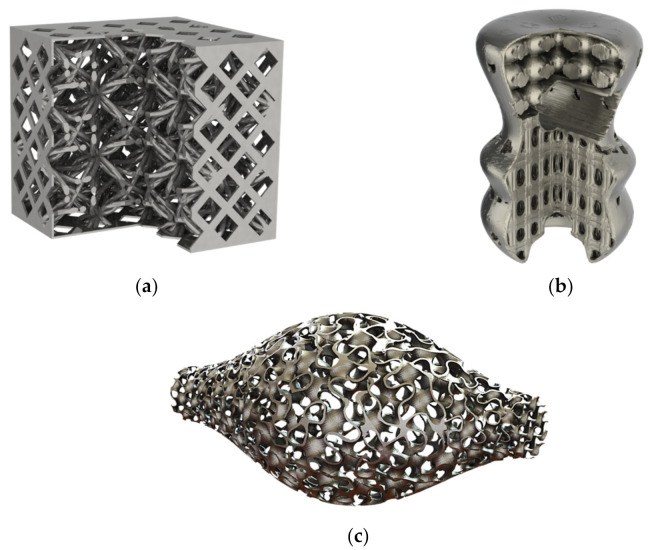
Shell generation and perforation: (**a**) Cubic shell perforated with a diamond pattern and filled with the artistic unit cell, (**b**) spline-based revolved shell perforated with a circular pattern and filled with multi-domain sized graded unit cells, and (**c**) two-step size graded gyroid shell for a sinusoidal revolved shape.

The shell perforation and integration were tested using a simple cube as the base part, while its patterning tool was a diamond shape. The shell was merged with a lattice structure based on an artistically designed unit cell shown in Figure 1 and the final output part is shown in Figure 6a. The second test generated a shell for a spline-based revolved part with different curvature radii along its contour. The generated shell to be perforated with a circular-shaped patterning tool. This part allowed the testing of the global and local loop resolving algorithm implemented in the DGLS. The shell was then integrated with a multi-domain size graded lattice structure, as shown in Figure 6b. In both scenarios, the patterning position followed a linear function. In the cube scenario, the patterning was projected from the three planes, while in the spline-revolved part, the patterns were projected from two planes only.

The DGLS can generate a two-step size graded shell, which is achieved by generating a shell from the base part without applying any perforation then propagate a size graded unit cell through the shell. An example of a size graded gyroid shell was generated for the sinusoidal revolved part with no infill inside, as shown in Figure 6c. The shell offsetting showed success in producing a shell without local or global loops, as shown in Figure 6b. However, the patterning tool did not produce a good pattern with highly curved parts. A conformal patterning algorithm would be more suitable for the highly curved shapes. The two-step shell was more successful with the TPMS shells compared to the strut-based unit cells. Using a relatively thick shell compared to the unit cell size used is essential to produce a continuously connected two-step shell.

## 4. Dual Grading Experimental Investigation

Two experiments were designed to understand the effect of size grading, relative density grading, and dual grading on the modulus of elasticity, compressive strength, absorbed energy, and failure shapes. The first experiment is intended to investigate the lattice structure designs developed using the upper and lower limits of the grading parameters using a constant unit cell size and relative density without applying any grading. The second experiment investigates several grading designs using different size and relative density grading equations. The designed specimens were additively manufactured using stereolithography (SLA). A digital image correlation system was used to analyze each specimen’s developed strain under compression testing and capture the failure shape.

### 4.1. Lattice Structure Design

**Figure 7:**
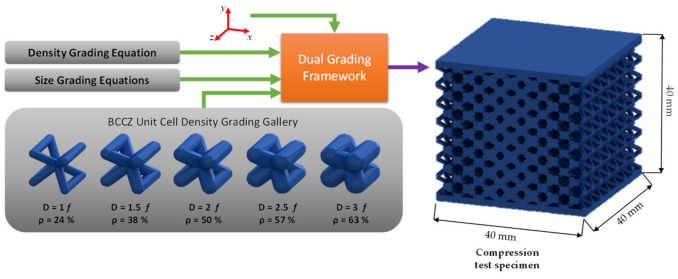
Generation of the dual graded lattice structure using a BCCZ grading gallery for compression test specimens.

The unit cell selected for the two experiments is the BCCZ unit cell. A discretized gallery consists of five BCCZ unit cells with different relative densities input to the dual grading framework and the spatial equations governing the cell and density grading, as shown in Figure 7. The dual graded experiment’s design envelope is defined by the selected upper and lower bounds of both the unit cell size and the unit cell relative density. The minimum relative density available in the gallery is 24%, with a strut diameter of 1 *f*, in which *f* is a scaling factor based on the cell size. The highest relative density is 63%, with a strut diameter of 3 *f*. Therefore, at a fixed cell size, the densest cell has a strut diameter of three times the lowest relative density cell. At a cell size of 10 × 10 mm, the lowest cell density has a 1 mm strut diameter, while the densiest unit cell has 3 mm strut diameter. The cell size is continuously varied using the size governing Equations (9)–(13) with a lower limit for any cell dimension, out of the three dimensions, is set to 3.5 mm, and the upper limit is set to 6.5 mm. The compression test specimen is a 40 × 40 × 40 mm^3^ cube, including a 2 mm thick shell at the top and the bottom of the designed lattice specimens, as shown in Figure 7, which follows the criteria specified in ISO 844:2014(E) [73]. The unit cells are propagated across the compression specimen domain with the orientation shown in Figure 7, in which the horizontal traverse strut is parallel to the top and bottom skin.

**Figure 8:**
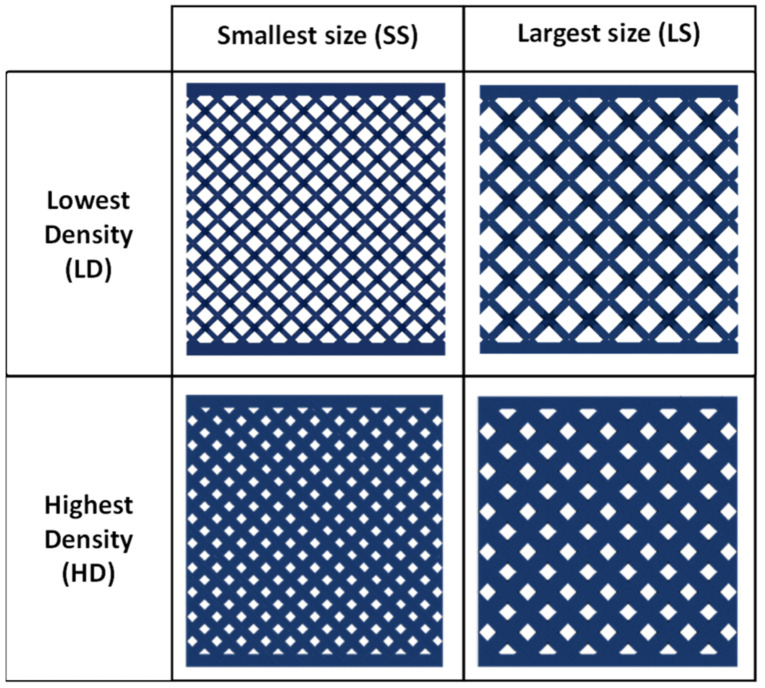
Front view of the boundary lattice structure designs for the compression test specimen.

The first experiment studies the effect of the unit cell’s size and relative density on the whole lattice structure’s mechanical properties. This experiment is considered a control experiment for the design domain boundary; the mechanical property values evaluated in this experiment are expected not to be exceeded by all the dual graded designs within the mentioned design envelop. The specimens in this experiment have a constant relative density and constant cell size unit cells. This experiment consists of four lattice structure designs using the upper and lower values of the relative density and size used in this investigation, as shown in Figure 8. The first boundary design has a 3.5 mm cell size and 24% relative density, which means that this design uses the smallest unit cell size (SS) and the lowest cell relative density (LD) of all the values used in this investigation. The second boundary design has the largest cell size (LS), of 6.5 mm, while having the lowest cell relative density (LD) used in this investigation. The third has the smallest cell size and the highest cell relative density (HD) of 63%. The fourth design has the largest cell size (LS) and the highest relative density (HD).

**Figure 9:**
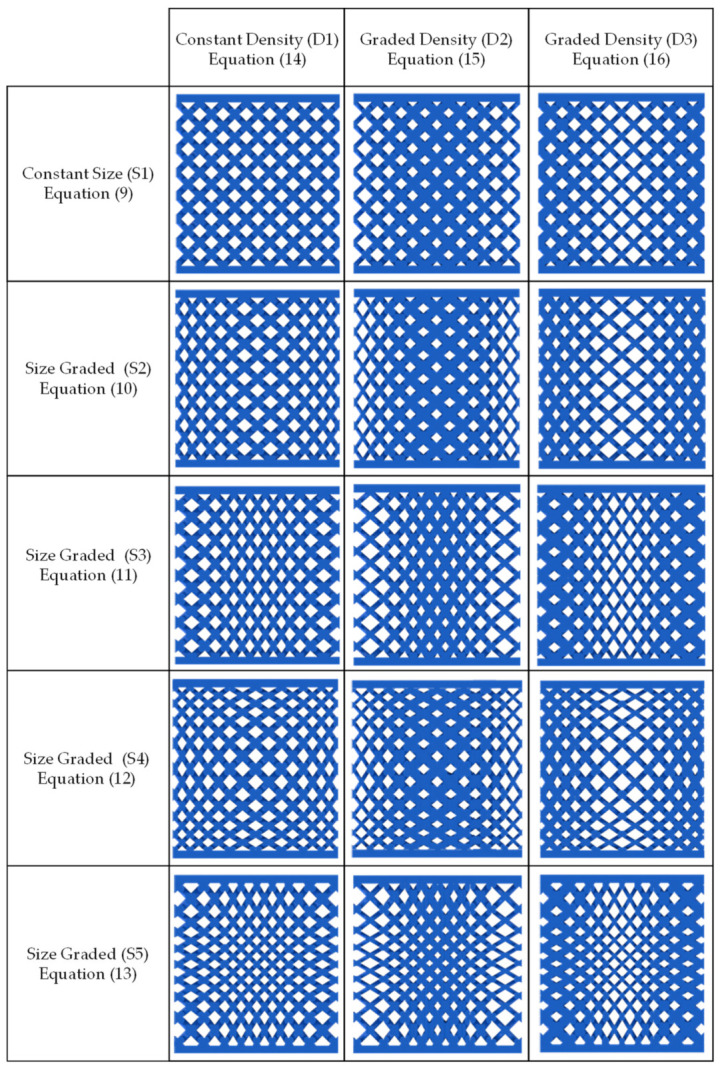
Front view of the dual graded lattice structure designs for compression test specimens.

The second experiment consists of fifteen unique lattice structure designs based on a full factorial experimental design, as shown in Figure 9. The experiment has two parameters. The first one represents the size grading governing equation, and the second one represents the relative density grading governing equation. The size grading parameter has five levels or grading equations as follows:

(9) $$S_{x_{\left(i,j,k\right)}}=C_{S};\;S_{y_{\left(i,j,k\right)}}=C_{S};\;S_{z_{\left(i,j,k\right)}}=C_{S}$$

where the first set of size grading equations (9) have a constant size unit cell distribution across the domain with the $C_{S}$ value that equals 5.5 mm. This value was chosen based on the average cell size used in other graded lattice structure designs. The origin of the Cartesian coordinate system is defined at the center of the mass of the cube. The size grading design described by (9) will be referred to as S1.

The second set of size grading equations (10),

(10) $$S_{x_{\left(i,j,k\right)}}=6.5-0.185x_{\left(i,j,k\right)};\;S_{y_{\left(i,j,k\right)}}=C_{S};S_{z_{\left(i,j,k\right)}}=6.5-0.185z_{\left(i,j,k\right)}$$

describe a size grading design with the largest unit cell around the origin, and the unit cell width decreases along the *x* and *z* axes towards the cube edges while the cell height is kept constant at the value $C_{S}$ along the *y*-axis. This size grading design allows the cell aspect ratio to vary between 1:1 to 1:1.5 along the *x* and *z* axes. The size grading design described by (10) will be referred to as S2. The third grading equation set (11),

(11) $$S_{x_{\left(i,j,k\right)}}=0.3x_{\left(i,j,k\right)}+3.5;\;S_{y_{\left(i,j,k\right)}}=C_{S};\;S_{z_{\left(i,j,k\right)}}=0.3z_{\left(i,j,k\right)}+3.5,$$

has the opposite grading direction of (10). The cell width starts with the smallest values at the middle of the cube and increases towards the cube edges along the *x* and *z* axes. Equation (11) also reverse the aspect ratio variation done by Equation (10). The size grading design described by (11) will be referred to as S3.

The fourth and fifth size grading designs are described by sets, Equations (12) and (13),

(12) $$S_{x_{\left(i,j,k\right)}}=6.5-0.185x_{\left(i,j,k\right)};\;S_{y_{\left(i,j,k\right)}}=6.5-0.185y_{\left(i,j,k\right)};\;S_{z_{\left(i,j,k\right)}}=6.5-0.185z_{\left(i,j,k\right)}$$

(13) $$S_{x_{\left(i,j,k\right)}}=0.3x_{\left(i,j,k\right)}+3.5;\;S_{y_{\left(i,j,k\right)}}=0.3y_{\left(i,j,k\right)}+3.5;\;S_{z_{\left(i,j,k\right)}}=0.3z_{\left(i,j,k\right)}+3.5$$

describe a three-dimensional size grading spatially. The design of Equation (12) starts with a large unit cell size in the middle of the cube, and it decreases towards the cube edges along the *x*, *y*, and *z* axes. The size grading design described by (12) will be referred to as S4. While Equation (13) has the smallest unit cell in the middle of the cube, and its size increases along the three axes towards the edges. The size grading design described by (13) will be referred to as S5. The aspect ratio of both the (S4) and (S5) designs vary along the three axes. However, the cells along the planar and spatial diagonals have a 1:1 aspect ratio. The geometry produced by the different size grading equation sets can be seen independently of any relative density grading in the first column of Figure 9.

The relative density grading designs are governed by three grading equations. The first Equation (14),

(14) $$\rho_{\left(i,j,k\right)}=C_{D}$$

represents a constant relative density across the specimen domain with a value of $C_{D}$ which equals 38% relative density. The density grading design described by (14) will be referred to as D1. The second Equation (15),

(15) $$\rho_{\left(i,j,k\right)}=-0.0485x_{\left(i,j,k\right)}^{2}-0.7044x_{\left(i,j,k\right)}+63$$

represents a relative density grading with a quadratic distribution. The highest relative density is in the middle of the cube and decreases along the *x*-axis towards the edges. The density grading design described by (15) will be referred to as D2. The third Equation (16),

(16) $$\rho_{\left(i,j,k\right)}=-0.0496x_{\left(i,j,k\right)}^{2}+2.8545x_{\left(i,j,k\right)}+24$$

represents a relative density grading along the *x*-axis, starting with the lowest relative density in the middle of the cube and increasing towards the cube edges. The density grading design described by (16) will be referred to as D3. The geometries produced by grading the relative density independently of the cell size grading are shown in the first row of Figure 9. The remaining designs in Figure 9 are the combination between the size and the relative density grading equations. All the lattice designs are fully symmetric about the three principal axes, with the center of the cube coinciding with the intersection point of all the symmetry planes.

### 4.2. Specimen Manufacturing

The specimens were manufactured using a Form 2 3D printer, Formlabs, MA, USA, a Stereolithography (SLA) desktop 3D printer. The material used is clear resin by Formlabs. The manufacturing parameters were chosen based on the recommended optimum parameters to produce high strength with moderate ultimate strain, as concluded by Garcia et al. [74]. The post-curing duration is the most significant parameter, with one hour as the optimum level. The test specimens were manufactured at 100 µm layer thickness and oriented with the side face of the lattice specimen facing down, and the skin is parallel to the manufacturing direction. The specimens were washed with isopropyl alcohol for 10 min and left to dry for one hour in a dark area. The total post-curing duration was one hour, distributed equally on all the lateral faces. Two replicas were manufactured for each lattice design to establish confidence while investigating a wide range of grading designs with nineteen unique lattice designs.

### 4.3. Compression Test and Digital Image Correlation

Compression tests were carried out using INSTRON 5966, Instron, MA, USA, universal testing systems for most specimens except six designs that exceeded 10 kN loading force. These were tested on an MTS 810 with 100 kN loading force capacity. The compression test was run at a loading speed of 1 mm/min without preloading, as recommended by ISO 604:2002(E) [75]. The compressive strength and the elastic modulus were determined based on the ISO standard criteria for rigid cellular plastic materials, ISO 844:2014(E) [73]. The compressive strength value equals the maximum compressive stress occurring before 10% compressive strain. Otherwise, it is equal to the stress corresponding to 10% compressive strain. The absorbed energy is not described in the previous standard. However, the ISO standard for cellular metal material, ISO 13314:2011(E) [76], describes it. The absorbed energy was calculated as the area under the stress-strain curve till the densification or 50% compressive strain. A failure is considered if the stress dropped 40% within a short time or after the compressive strain reaches 40%.

A stereo digital image correlation system (DIC) was used to capture and analyze the different lattice structure designs’ deformation during compression testing. The system consists of two Pike F421b cameras, Allied Vision Technology, PA, USA, with a 2048 × 2048 pixels CCD sensor. Each camera is equipped with a 28–85 mm AF macro zoom lens, Nikon, Tokyo, Japan, with adjustable aperture, zoom, and focus separately. Before testing each specimen, the three lens parameters were adjusted so that the specimen is in focus and with balanced lighting across the specimen. The magnification range across all the specimens was between 9 to 14 pixels/mm. The angle between the two cameras was set to 25°, with one of the cameras looking perpendicular to the specimen’s front face.

Testing was captured at one frame per second, which means that each frame was capturing a compressive displacement of around 20 µm based on the loading rate of 1 mm/min. The specimens were sprayed with black and white spray paint to create a scattered speckle pattern on the lattice specimen’s front face. Before each test, a DIC calibration process was carried out by capturing a calibration pattern consisting of 8 × 8 circular dots with a 4 mm diameter at several spatial rotations. The calibration and the test images were captured using VIC-Snap software, V7.0, Correlated Solutions, SC, USA. Moreover, the image processing and data analysis were performed using VIC-3D software by Correlated Solutions. The average calibration score was around 0.06, with a maximum value of 0.08 and a minimum value of 0.04. The subset was tuned for each specimen to minimize the projection error; the maximum subset value was 67 × 67 pixels, and the minimum value was 35 × 35 pixels. The step value was set to 11 pixels. The average projection error equals 0.1, with a maximum value of 0.46 and a minimum value of 0.021.

### 4.4. Results

#### 4.4.1. Mechanical Properties

**Figure 10:**
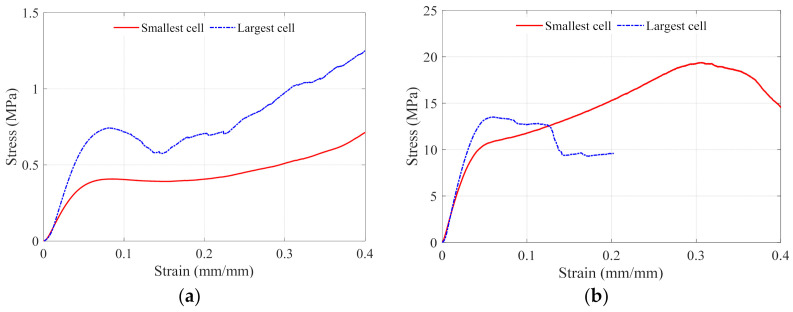
Stress-strain curves for different cell sizes at (**a)** LD and (**b**) HD.

The boundary lattice structure design experimental results are presented in Table 1. The relative density of the unit cell has the most significant influence on the compressive strength. The increase in the unit cell relative density from 24% to 63% leads to double the increase in the whole specimen volume ratio, which is the ratio between the lattice structure volume and the occupied solid volume domain. The average strength and modulus of elasticity increased over 20 times. The stress-strain curves for the boundary-designed lattice structure using the lowest and highest unit cell relative density are shown in Figure 10a,b, respectively. Comparing these two figures, as the cell size increases at the same relative density, the compressive strength and the modulus of elasticity increase. The increase in the mechanical properties values is comparatively high at lower unit cell relative density. However, at higher relative densities, the mechanical properties values do not increase significantly.

On the other hand, as cell size increases at both the low and high relative density, the compressive strain decreases to be less than 10%. Small cell size resulted in a smooth and steady compressive stress-strain curve without sudden stress drops for either the LD or HD designs. The large cell size resulted in a significant stress drop just after a 10% compressive strain, and in the case of the LD design, densification occurred, and the stress increased with frequent stress drops. In contrast, in the HD design case, a complete failure occurred during the first stress drop. The lowest modulus of elasticity and compressive strength in the boundary designs belongs to the LDSS lattice structure and equals 10.53 MPa and 0.42 MPa, respectively. The highest modulus of elasticity and compressive strength were 368.40 MPa with 13.52 MPa, respectively, for the HDLS lattice. The absorbed energy had a reversed response at the low and high relative density designs. As the cell size increases, so the absorbed energy increases at the LD designs. However, in the HD designs, as the cell size increases, the absorbed energy decreases. The LDSS lattice has the lowest energy absorption found, and it is equal to 0.12 MJ/m^3^, while the HDSS has the highest and equals 3.94 MJ/m^3^.

**Figure 11:**
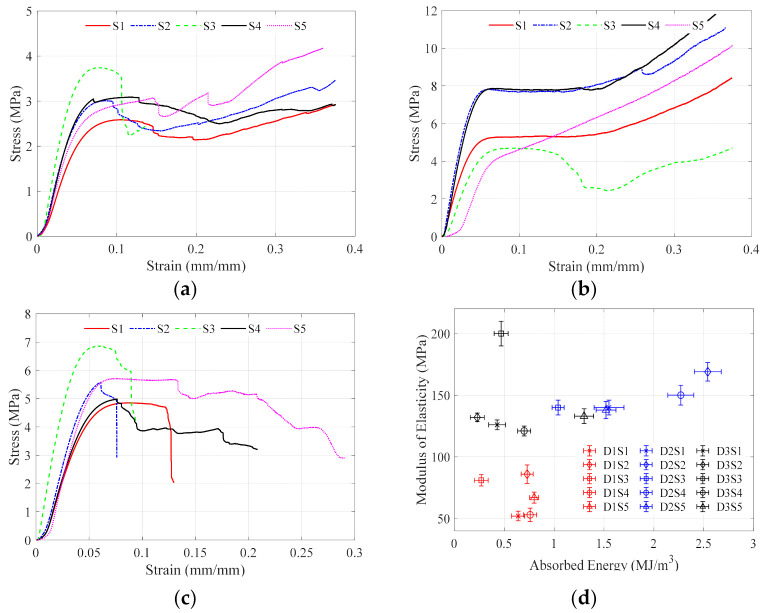
Stress-strain curves for (**a**) different cell size grading designs at D1, (**b**) different cell size grading designs at D2, (**c**) different cell size grading designs at D3, and (**d**) the modulus of elasticity and absorbed energy versus the 15 graded lattice designs.

In the dual graded design experiment, the stress-strain curves’ general profiles and the mechanical properties values of these lattices are classified into three main groups based on their relative density grading function group. The mechanical properties values, compressive plateaus, and volume ratios vary within each group’s specific range. For the first group, D1, which has a constant relative density, the size grading did not affect the volume ratio of the test specimen, which is constant for all the size graded designs with a value of 0.53, as shown in Table 2. By observing the stress-strain curves shown in Figure 11a, all the size graded designs have a similar trend, which after the maximum stress value is reached, a small stress drop occurs. The stress starts to increase except for design S3, which ultimately fails after reaching the maximum stress value. This group’s modulus of elasticity ranges between 51.60 MPa (S1) to 86.10 MPa (S2), while the compressive strength is almost 3.1 MPa for all the specimens except S1. The compressive strain is set to 10% from all samples except S2 and S3, which occurs around 7%. The energy absorption is the only parameter with a significant variation by changing the size grading as it ranges between 0.27 MJ/m^3^ (S3) to 0.80 MJ/m^3^ (S5). In this group, the size grading significantly controlled the absorbed energy and the modulus of elasticity. It was shown that design S3 is a brittle design. However, it has high strength and elasticity. The S1, which has no grading in size or relative density, shows the lowest modulus of elasticity and compressive strength and average energy absorption. S4 and S5 designs have the highest energy absorption.

The stress-strain curves of the second-relative density graded group D2, shown in Figure 11b, are generally smoother curves with steady compressive plateaus and no stress drops. This group’s average compressive strength is higher than the two other groups, with a maximum of 8.10 MPa (S2) and a minimum of 5.40 MPa (S1). The modulus of elasticity is around 140 MPa for most samples except S2, which has the highest value of 169.20 MPa. Only the S3 design reached the compressive strain at 5%, and a significant stress drop occurred afterwards. Almost all the samples had a compressive plateau after reaching the maximum stress value. These plateaus occurred between 10% to 15% strain, except for S5, which started to increase directly after the elastic zone region. The average energy absorption is higher for all the D2 groups than the two other groups, with the highest at 2.54 MJ/m^3^ (S2) and the lowest at 1 MJ/m^3^ (S3). The volume ratio is around 0.6 except for S3 and S5, which are at 0.55. S2 and S4 designs pose the highest material properties in the D2 group. Within this group, the size grading designs noticeably affect the mechanical properties.

**Figure 12:**
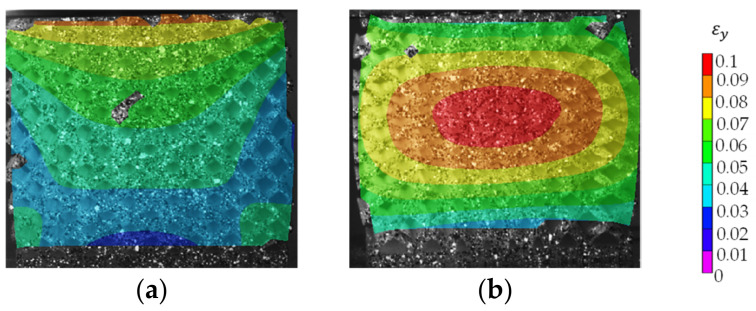
Digital Image Correlation (DIC) results depicting the two common engineering strain distribution patterns in the vertical direction at 10% compressive strain (**a**) unidirectional and (**b**) concentric strain distributions pattern.

The last group is D3, which has a generally brittle profile with low elasticity. As shown in Figure 12, the stress-strain curves have a significant stress drop with a complete failure after reaching the maximum stress value. Only two size-graded designs, S1 and S5, reached a compressive plateau after reaching the maximum stress value before complete failure; S5 showed a better stress-strain curve than S1 as it is the only specimen that failed at 30% strain. The D3 group has a higher average compressive strength and modulus of elasticity than the D1 group but is still lower than the D2 group. However, the average energy absorption of this group is lower than the D1 group and D2 group. The volume ratio of this group for most of the specimens is 0.55. The modulus of elasticity is 199.6 MPa for S3, and the lowest is 121.2 MPa for S4. The compressive strength variation due to size grading was not substantial as the highest compressive strength is 5.70 MPa for S3 while the lowest is 4.70 MPa for both S1 and S2. Most specimens in this group reached the maximum stress at around 6% strain, except S1 and S5, which reached the maximum stress at 10%. S5 design achieved the highest absorbed energy with a value of 1.3 MJ/m^3^, and the lowest value was 0.23 MJ/m^3^ for the S2 design.

By looking at Figure 11d, it can be easily noticed that each density grading design has its region defined by the modulus of elasticity and the amount of absorbed energy. The first region occupied by the red marker is for the D1 designs, the second region is occupied by the blue markers and is for the D2 design, and the third region is occupied by the black markers and is for the D3 design, which indicates the significance of the density grading on both parameters. D2 design has the highest obtained values for all the mechanical properties compared to the D1 and D3. Within each relative density grading group, the unit cell size grading can fine-tune and maximize the mechanical properties and improve the stress-strain curve. Additionally, there is an interaction between both density grading designs and the size grading designs. S2 design maximized the modulus of elasticity and the compressive strength in both D1 and D2 design groups. S4 and S5 designs maximized the energy absorption within D1 and D3 design groups. S2 and S4 maximized the energy absorption within the D2 group. S3 design produced a brittle mechanical performance with a complete failure under a small amount of strain for all three design groups. D2S2 design is considered the best design followed by D2S4 to achieve the highest absorbed energy and ultimate tensile strength.

#### 4.4.2. Deformation Behavior

Digital image correlation was used to understand each lattice structure’s deformation behavior. The deformation was evaluated for the specimen’s front surface only. All of the distributions are rendered at the compressive strength and strain. The DIC results are stable within a range of deformation of 10% strain value. Above 10% strain, the strain analysis either fails to evaluate the distribution or produces high error renders. The vertical direction’s engineering strain has two distinctive distribution patterns, as shown in Figure 12a,b. Pattern (a) has the maximum strain at the top of the specimen and decreases towards the specimen’s base. Pattern (b) has concentric strain distribution with maximum value in the center, and it decreases towards the edges of the specimen. Pattern (b) is the dominant distribution for most specimens, while distribution (a) occurred in specimen LDLS, D3S2, D3S3, D3S4, and D1S3. Pattern (a) occurred three times in group D3 and two times in group S3.

**Figure 13:**
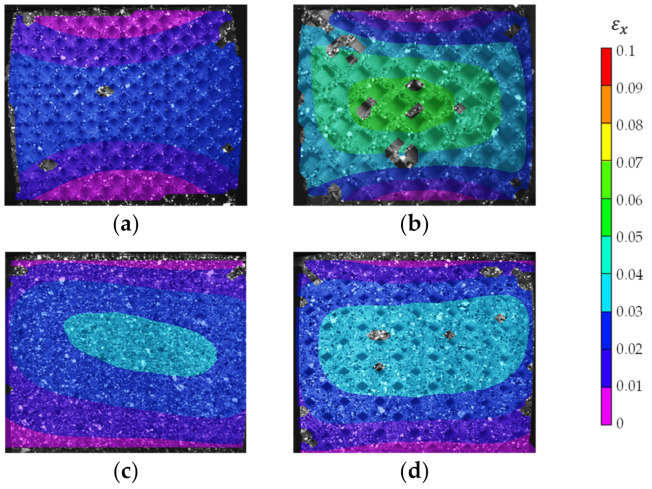
Digital Image Correlation (DIC) results of the boundary lattice designs depicting the engineering strain distribution in the horizontal direction at 10% compressive strain for (**a**) LDSS, (**b**) LDLS, (**c**) HDSS, and (**d**) HDLS boundary designs.

**Figure 14:**
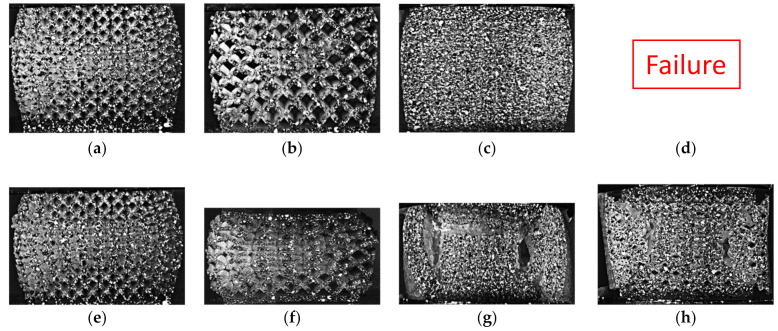
Deformation of the boundary lattice designs at 30%: (**a**) LDSS, (**b**) LDLS, and (**c**) HDSS, (**d**) HDLS, and deformation of the boundary lattice designs at the end of the test: (**e**) LDSS, (**f**) LDLS, (**g**) HDSS, and (**h**) HDLS.

Engineering strain in the horizontal direction is evaluated for each design to investigate the different designs’ deformation behavior. For the low relative density (LD) boundary designs, at 10% compressive strain, the horizontal engineering strain distribution is symmetric for both the LDSS and the LDLS, as shown in Figure 13a,b, respectively. The LDLS tends to have a concentric distribution with a high engineering strain in the middle and decreases towards the edges. The LDSS has a hyperbolic distribution. The LDLS had a unidirectional compressive strain shown in Figure 12a and had a noticeable stress drop after the maximum stress value shown in the stress-strain curve (Figure 10a). By analyzing the part’s deformation beyond 10% strain, it was observed that there was a 45° initiated crack from the right-top corner as shown in Figure 14b, and unit cells were compressed along that line. For the LDSS design, the cells were squeezed in the middle horizontally, as shown in Figure 14a. At the end of the test, the LDSS design retrieved most of its original height and width, while the LDLS was fractured and remained in its compressed state as shown in Figure 14e,f, respectively.

The vertical and horizontal engineering strain distribution shows a concentric pattern for the high relative density boundary designs, as shown in Figure 13c,d. The HDSS specimen was observed to be intact with slight buckling at 30% strain, as shown in Figure 14c. Both HD designs were fractured entirely at the end of the test, with a complete separation of the sidewalls and large vertical cavities appearing in the middle of the part, as shown in Figure 14g,h.

**Figure 15:**
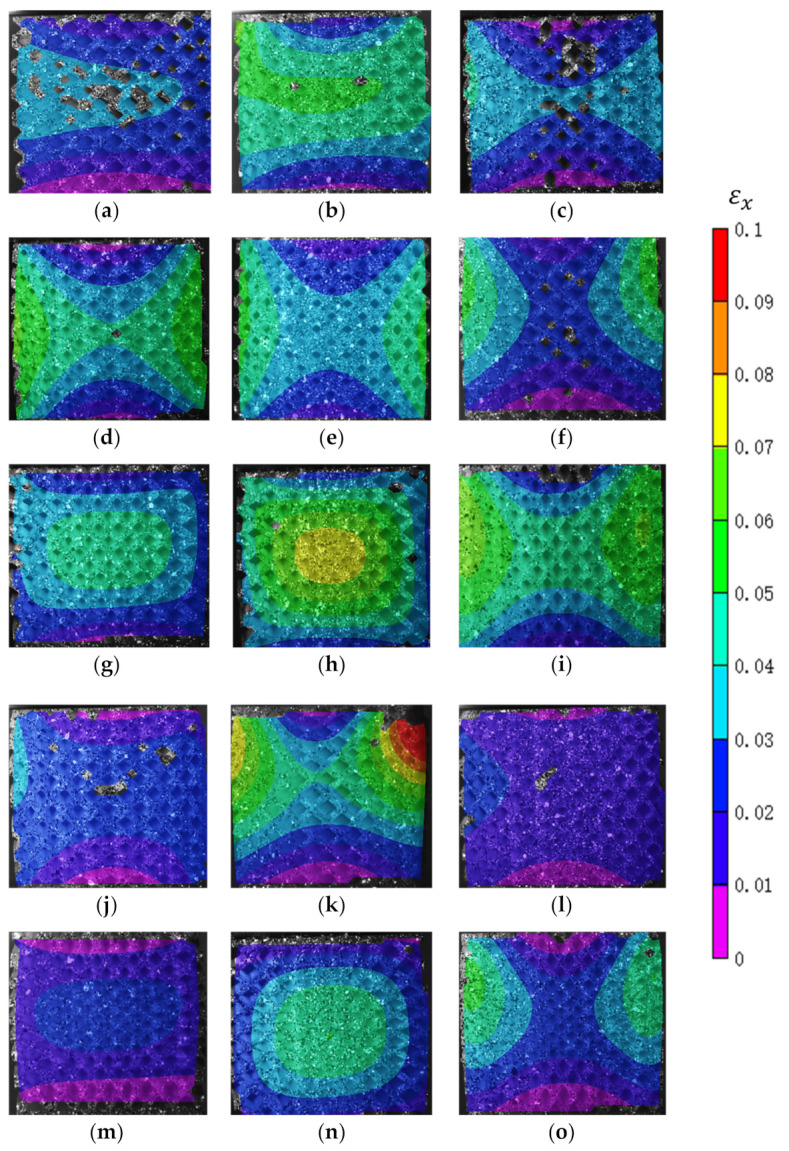
Digital Image Correlation (DIC) results of the functionally graded lattice designs depicting the strain distribution in the horizontal direction at 10% compressive strain: (**a**) D1S1, (**b**) D2S1, (**c**) D3S1, (**d**) D1S2, (**e**) D2S2, (**f**) D3S2, (**g**) D1S3, (**h**) D2S3, (**i**) D3S3, (**j**) D1S4, (**k**) D2S4, (**l**) D3S4, (**m**) D1S5, (**n**) D2S5, and (**o**) D3S5.

For the dual graded designs, at 10% compressive strain, most of the designs followed a concentric distribution for the vertical strain distribution. However, six designs had a unidirectional distribution, namely, D1S3, D3S3, D3S2, D2S4, D3S4, and D1S5. For the horizontal strain distribution obtained at 10% compressive strain, two main patterns were found. The first pattern is the concentric pattern, and the second pattern follows a hyperbolic distribution, as shown in Figure 15. Most of the dual graded lattice designs followed a hyperbolic distribution, while only the D1S3, D2S3, D1S5, and D2S5 had a concentric distribution. The peak strain is in the center within the concentric distribution. The average peak value at 10% compressive strain was 0.4, while the maximum engineering strain recorded was 0.7 and belonged to the D2S3. On the other hand, the hyperbolic distribution has its peak engineering strain at the side edges of the specimen, and the average peak value was 0.45, and the highest was 0.85 and belonged to the D2S4.

**Figure 16:**
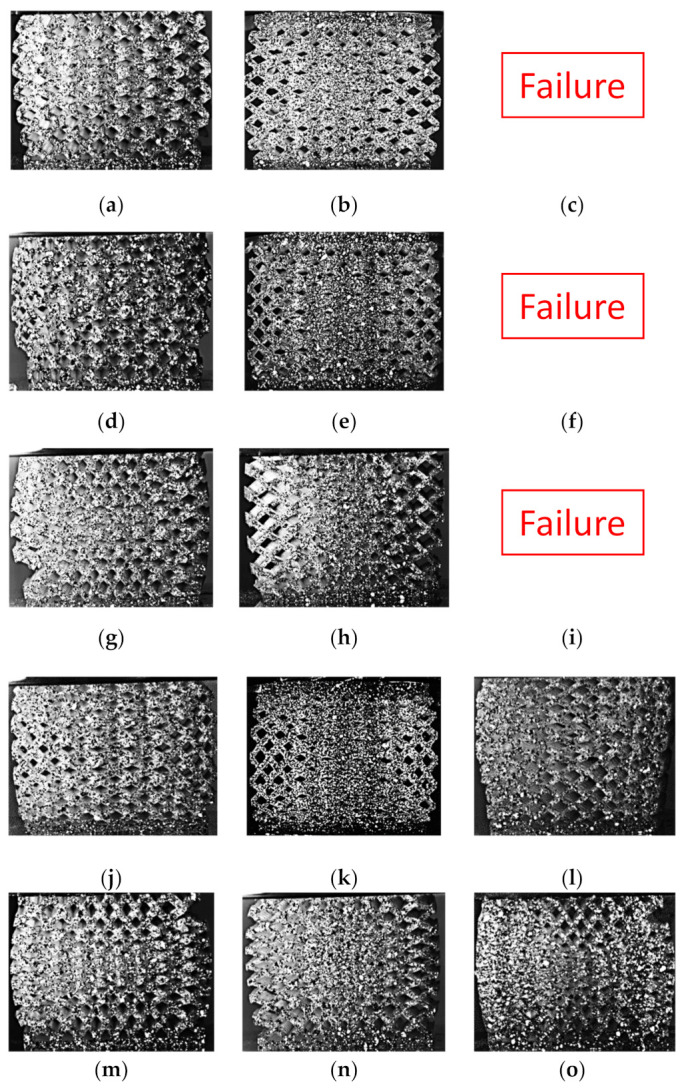
Deformation of the dual graded lattice designs at 30% compressive strain: (**a**) D1S1, (**b**) D2S1, (**c**) D3S1, (**d**) D1S2, (**e**) D2S2, (**f**) D3S2, (**g**) D1S3, (**h**) D2S3, (**i**) D3S3, (**j**) D1S4, (**k**) D2S4, (**l**) D3S4, (**m**) D1S5, (**n**) D2S5, and (**o**) D3S5.

The deformation shape at 30% compressive strain was captured and presented in Figure 16, but the DIC analysis failed at this large deformation. The first three size grading designs at the third relative density grading design (D3) failed before reaching 30% compressive strain. Three designs showed partial fracture D1S3, D1S5, D2S1, and D3S5. A visible crack initiated from the corners of D1S5 and D2S1. Most of the buckling shapes found were round, with the highest displacement in the middle of the buckling curvature. Only two designs, D1S2 and D3S4, showed a different buckling shape where the buckling displacement was more significant at the top of the lattice than the lower part. The D2 designs showed higher densification in the middle of the lattice specimen than all the other lattices.

**Figure 17:**
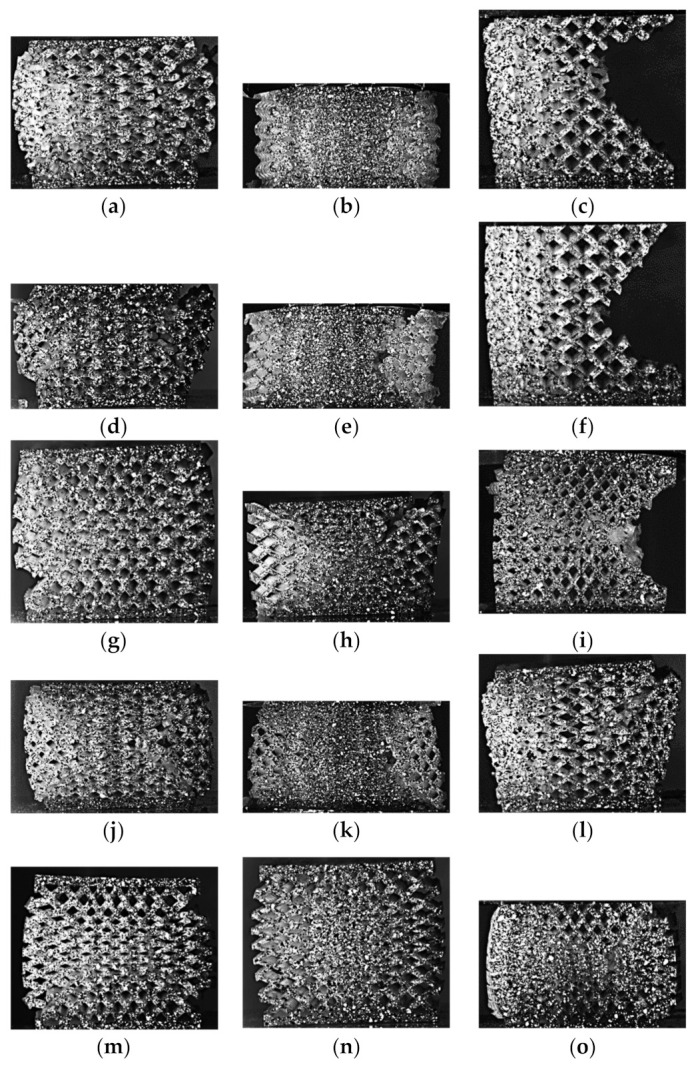
Deformation of the functionally graded lattices at the end of the compression test: (**a**) D1S1, (**b**) D2S1, (**c**) D3S1, (**d**) D1S2, (**e**) D2S2, (**f**) D3S2, (**g**) D1S3, (**h**) D2S3, (**i**) D3S3, (**j**) D1S4, (**k**) D2S4, (**l**) D3S4, (**m**) D1S5, (**n**) D2S5, and (**o**) D3S5.

The failure and fracture shape of the specimens after the compression tests are shown in Figure 17. For the D1 designs, the S2 designs showed the most crack growth, and the crack was initiated diagonally from two orthogonal directions. Both S1 and S4 were fractured diagonally across the whole lattice, and the struts were broken. Both S3 and S5 showed a minor fracture from the side surfaces, and they retained a significant portion of their original height afterwards. For the D2 designs, the failure shapes show complete specimen densification. The crack initiated near the lattice’s edges, with the middle part of the lattice still intact. The S5 design did not show any signs of significant cracks, and it restored a significant portion of its original height. The first three size grading designs for the D3 designs have a common fracture pattern. The fracture shape is a large triangular segment of around 20% to 35% of the lattice volume. The triangular segment was ultimately separated and flew away forcefully from the part. The 2D size grading seems to improve the fracture behavior and the elongation in S4 and S5 designs. The S5 designs seem to improve the lattice’s overall integrity despite the relative density grading design used.

## 5. Conclusions

In this paper, a dual graded lattice structure generation framework is introduced. The developed framework was tested against several types of unit cell lattices, such as strut-based, triply periodic minimal surfaces, topology-optimized, and artistically designed unit cells and showed a promising performance. Several size grading and dual grading equations and their axes dependency were investigated. The framework utilized a discrete unit cell grading gallery to efficiently manipulate the unit cells’ relative density and porosity while grading the unit cells’ size. The effect of cell size, relative density, and dual grading on the mechanical properties, deformation behavior, and fracture shape was investigated experimentally.

For non-graded lattices, as the unit cell size increases, the compressive strain decreases. As the unit cell size decreases, the stress-strain curve becomes smooth and steady with a higher compressive strain. A slight increase in the unit cell’s relative density increases the compressive strength and the absorbed energy significantly. As the unit cell relative density decreases, the resistance to fracture increases and the part’s ability to restore its original shape increases.

The relative density grading function sets the average values for the compressive strength, modulus of elasticity, and absorbed energy for the dual graded lattices. In contrast, the unit cell’s size grading function would fine-tune the mechanical properties and stress-strain curve. By comparing the average compressive strength values achieved by the three relative density grading functions used in this study, it was found that the constant relative density has the lowest average compressive strength of 2.9 MPa at an average volume ratio of 0.53. The second relative density grading function achieves the highest average compressive strength of 6.8 MPa at an average volume ratio of 0.58. The third relative density grading function has an average compressive strength of 5 MPa at an average volume ratio of 0.57. Even normalizing the compressive strength values using the corresponding volume ratio, the normalized compressive strength values are 5.4 MPa, 11.7 MPa, and 8.7 MPa for the first (D1), second (D2), and third (D3) relative density grading functions, respectively. The relative density grading function affects the stress-strain curve significantly. The D2 lattices have a steady and smooth stress-strain curve, in which the stress value increases after reaching the compressive strength value without any sudden stress drops compared to the D1. D3 lattices have a general brittle behavior and break right after reaching the compressive strength with compressive strain below 10%. The D2 lattices have the highest average normalized absorbed energy (divided by the volume ratio) of 3 MJ/m^3^ compared to D1 and D3 lattices with around 1.2 MJ/m^3^.

The size grading functions fine-tune the absorbed energy value and the deformation and fracture behavior. The size grading did not affect the compressive strength in the constant relative density lattices (D1) and the graded density lattices (D3) significantly; however, its tuning effect was significant in the D2 relative density graded lattices. For the D2 relative density graded lattices, the size grading function S2 increased the compressive strength to 8 MPa from the 5.4 MPa achieved by the S1 lattice, which has a constant unit cell size; both designs have the same volume ratio of 0.6. The absorbed energy also increased to 2.54 MJ/m^3^ achieved by the S2 lattice from the 1.04 MJ/m^3^ achieved by the S3 lattice. The S3 size grading function generally introduces brittleness irrespective of the density grading function. However, the S5 size grading function increases the lattice’s fracture resistance and allows the lattice to endure higher compressive strains without showing significant fractures. It was also noticed that the S5 lattices would return to roughly their original height after the compression test compared to other size graded lattices. The dual grading combination that produced the highest compressive strength and absorbed energy is the D2S2 design. The D2S5 design has the highest fracture resistance among all designs. The D3S3 has the highest elastic modulus and the lowest compressive strain.

The dual graded lattice structure framework (DGLS) currently has some limitations. One of the limitations is that the unit cell’s spatial rotation is fixed throughout the lattice structure. Additionally, the DGLS can vary the relative density along one axis only. The future suggested upgrades are to implement the conformal algorithm to allow the cell orientation to improve the trimming of the cells at the part boundary; this will allow for conformal perforation of the surface. The relative density should be graded as a function of the three axes and not just one.

## Figures and Tables

**Table 1:** The results for the average mechanical properties of boundary lattice structure designs.

Design Configurations		Modulus of Elasticity (MPa)	Compressive Strength (MPa)	Compressive Strain (%)	Absorbed Energy (MJ/m^3^)	Volume Ratio
LD	SS	10.50 ± 1.90	0.40 ± 0.05	10	0.12 ± 0.08	0.36
	LS	18.70 ± 3.40	0.80 ± 0.03	8	0.20 ± 0.12	0.36
HD	SS	330.30 ± 22.30	11.70 ± 1.30	10	3.90 ± 0.20	0.73
	LS	369.60 ± 28.70	13.50 ± 1.60	5.5	1.50 ± 0.13	0.71

**Table 2:** The results for the average mechanical properties of dual graded lattice structure designs.

Design Configurations		Modulus of Elasticity (MPa)	Compressive Strength (MPa)	Compressive Strain (%)	Absorbed Energy (MJ/m^3^)	Volume Ratio
D1	S1	51.60 ± 7.80	2.30 ± 0.60	10	0.64 ± 0.13	0.53
	S2	86.10 ± 14.70	3.10 ± 0.10	8	0.73 ± 0.12	0.53
	S3	81.50 ± 9.60	3.00 ± 0.70	6	0.27 ± 0.08	0.53
	S4	52.90 ± 11.10	3.10 ± 0.80	10	0.76 ± 0.13	0.53
	S5	67.50 ± 8.60	3.10 ± 0.06	10	0.80 ± 0.09	0.53
D2	S1	139.30 ± 12.40	5.50 ± 0.30	10	1.55 ± 0.30	0.60
	S2	169.20 ± 14.70	8.10 ± 0.40	10	2.54 ± 0.27	0.60
	S3	139.70 ± 12.20	5.70 ± 0.60	5	1.00 ± 0.12	0.55
	S4	150.00 ± 15.80	7.80 ± 0.30	10	2.27 ± 0.26	0.60
	S5	138.50 ± 13.60	5.90 ± 1.30	10	1.52 ± 0.20	0.55
D3	S1	126.50 ± 7.90	4.70 ± 0.30	10	0.43 ± 0.17	0.55
	S2	131.80 ± 6.80	4.70 ± 0.80	6	0.23 ± 0.14	0.55
	S3	199.60 ± 20.00	5.80 ± 1.10	5	0.47 ± 0.10	0.60
	S4	121.20 ± 8.30	4.50 ± 0.70	7	0.70 ± 0.13	0.55
	S5	133.50 ± 11.80	5.20 ± 0.50	10	1.30 ± 0.20	0.60

## Data Availability

The STL files used in this study are openly available at Mendeley Data, Elsevier, doi: 10.17632/n38x2tfzk7.1, [77].

## References

[1] Bose S., Bandyopadhyay A. (2019). Additive Manufacturing.

[2] Aguilera A.F.E., Nagarajan B., Fleck B.A., Qureshi A.J. (2019). Ferromagnetic particle structuring in material jetting—Manufacturing control system and software development. Procedia Manuf..

[3] Gibson I., Rosen D., Stucker B. (2015). Additive Manufacturing Technologies.

[4] Hertafeld E., Zhang C., Jin Z., Jakub A., Russell K., Lakehal Y., Andreyeva K., Bangalore S.N., Mezquita J., Blutinger J. (2019). Multi-material three-dimensional food printing with simultaneous infrared cooking. 3D Print. Addit. Manuf..

[5] Singhal T., Khare A., Gupta N., Gandhi T.K. 3D-printed sole with variable density using foot plantar pressure measurements. Proceedings of the 2018 IEEE 8th International Advance Computing Conference (IACC).

[6] Omiyale B.O., Farayibi P.K. (2020). Additive manufacturing in the oil and gas industries. Analecta Tech. Szeged..

[7] Paolini A., Kollmannsberger S., Rank E. (2019). Additive manufacturing in construction: A review on processes, applications, and digital planning methods. Addit. Manuf..

[8] Rupal B.S., Anwer N., Secanell M., Qureshi A.J. (2020). Geometric tolerance and manufacturing assemblability estimation of metal additive manufacturing (AM) processes. Mater. Des..

[9] Rupal B.S., Mostafa K., Wang Y., Qureshi A.J. (2019). A Reverse CAD approach for estimating geometric and mechanical behavior of fdm printed parts. Procedia Manuf..

[10] Mostafa K., Qureshi A.J., Montemagno C. (2017). Tolerance Control using subvoxel gray-scale DLP 3D printing. ASME International Mechanical Engineering Congress and Exposition.

[11] Mostafa K.G., Arshad M., Ullah A., Nobes D.S., Qureshi A.J. (2020). Concurrent Modelling and experimental investigation of material properties and geometries produced by projection microstereolithography. Polymers.

[12] Blanco I. (2020). The use of composite materials in 3D printing. J. Compos. Sci..

[13] Gibson I. (2017). The changing face of additive manufacturing. J. Manuf. Technol. Manag..

[14] Helou M., Kara S. (2017). Design, analysis and manufacturing of lattice structures: An overview. Int. J. Comput. Integr. Manuf..

[15] Tosto C., Saitta L., Pergolizzi E., Blanco I., Celano G., Cicala G. (2020). Methods for the Characterization of polyetherimide based materials processed by fused deposition modelling. Appl. Sci..

[16] Cicala G., Giordano D., Tosto C., Filippone G., Recca A., Blanco I. (2018). Polylactide (PLA) Filaments a biobased solution for additive manufacturing: Correlating rheology and thermomechanical properties with printing quality. Materials.

[17] Mahmoud D., Elbestawi M.A. (2017). Lattice structures and functionally graded materials applications in additive manufacturing of orthopedic implants: A Review. J. Manuf. Mater. Process..

[18] Aremu A., Brennan-Craddock J., Panesar A., Ashcroft I., Hague R., Wildman R., Tuck C. (2017). A voxel-based method of constructing and skinning conformal and functionally graded lattice structures suitable for additive manufacturing. Addit. Manuf..

[19] Feng J., Fu J., Shang C., Lin Z., Niu X., Li B. (2020). Efficient generation strategy for hierarchical porous scaffolds with freeform external geometries. Addit. Manuf..

[20] Yang C., Xu K., Xie S. (2020). Comparative study on the uniaxial behaviour of topology-optimised and crystal-inspired lattice materials. Metals.

[21] Bonatti C., Mohr D. (2019). Mechanical performance of additively-manufactured anisotropic and isotropic smooth shell-lattice materials: Simulations & experiments. J. Mech. Phys. Solids.

[22] Nazir A., Abate K.M., Kumar A., Jeng J.-Y. (2019). A state-of-the-art review on types, design, optimization, and additive manufacturing of cellular structures. Int. J. Adv. Manuf. Technol..

[23] Kumar S., Tan S., Zheng L., Kochmann D.M. (2020). Inverse-designed spinodoid metamaterials. NPJ Comput. Mater..

[24] Wu J., Aage N., Westermann R., Sigmund O. (2018). Infill optimization for additive manufacturing—Approaching bone-like porous structures. IEEE Trans. Vis. Comput. Graph..

[25] Rana U.A., Koon T.W., Mostafa K., Baqai A.A., Qureshi A.J. Characterization of cuttlebone for adaptive infills. Proceedings of the 2017 8th International Conference on Mechanical and Aerospace Engineering (ICMAE).

[26] Chen Z., Xie Y.M., Wu X., Wang Z., Li Q., Zhou S. (2019). On hybrid cellular materials based on triply periodic minimal surfaces with extreme mechanical properties. Mater. Des..

[27] Xue Y., Wang X., Wang W., Zhong X., Han F. (2018). Compressive property of Al-based auxetic lattice structures fabricated by 3-D printing combined with investment casting. Mater. Sci. Eng. A.

[28] Liu J., Gaynor A.T., Chen S., Kang Z., Suresh K., Takezawa A., Li L., Kato J., Tang J., Wang C.C.L. (2018). Current and future trends in topology optimization for additive manufacturing. Struct. Multidiscip. Optim..

[29] Piper S. From the Lab: Auxetic Smart Structures—PIPER3DP. http://www.piper3dp.com/blogs/from-the-lab-auxetic-smart-structures/.

[30] Rahmani R., Antonov M., Kollo L., Holovenko Y., Prashanth K.G. (2019). Mechanical behavior of Ti6Al4V scaffolds filled with CaSiO3 for implant applications. Appl. Sci..

[31] Qin Y., Wen P., Guo H., Xia D., Zheng Y., Jauer L., Poprawe R., Voshage M., Schleifenbaum J.H. (2019). Additive manufacturing of biodegradable metals: Current research status and future perspectives. Acta Biomater..

[32] Zadpoor A.A. (2019). Mechanical performance of additively manufactured meta-biomaterials. Acta Biomater..

[33] Espin-Lopez P., Pasian M. Compact 3D-printed variable-infill antenna for snow cover monitoring. Proceedings of the 12th European Conference on Antennas and Propagation (EuCAP 2018).

[34] Thompson M.K., Moroni G., Vaneker T., Fadel G., Campbell R.I., Gibson I., Bernard A., Schulz J., Graf P., Ahuja B. (2016). Design for additive manufacturing: Trends, opportunities, considerations, and constraints. CIRP Ann..

[35] Dumas M., Terriault P., Brailovski V. (2017). Modelling and characterization of a porosity graded lattice structure for additively manufactured biomaterials. Mater. Des..

[36] De Aquino D.A., Maskery I., Longhitano G.A., Jardini A.L., Del Conte E.G. (2020). Investigation of load direction on the compressive strength of additively manufactured triply periodic minimal surface scaffolds. Int. J. Adv. Manuf. Technol..

[37] Cutolo A., Engelen B., Desmet W., Van Hooreweder B. (2020). Mechanical properties of diamond lattice Ti–6Al–4V structures produced by laser powder bed fusion: On the effect of the load direction. J. Mech. Behav. Biomed. Mater..

[38] Liu L., Kamm P., García-Moreno F., Banhart J., Pasini D. (2017). Elastic and failure response of imperfect three-dimensional metallic lattices: The role of geometric defects induced by Selective Laser Melting. J. Mech. Phys. Solids.

[39] Maconachie T., Leary M., Lozanovski B., Zhang X., Qian M., Faruque O., Brandt M. (2019). SLM lattice structures: Properties, performance, applications and challenges. Mater. Des..

[40] Abou-Ali A.M., Al-Ketan O., Lee D.-W., Rowshan R., Abu Al-Rub R.K. (2020). Mechanical behavior of polymeric selective laser sintered ligament and sheet based lattices of triply periodic minimal surface architectures. Mater. Des..

[41] Nagesha B., Dhinakaran V., Shree M.V., Kumar K.M., Chalawadi D., Sathish T. (2020). Review on characterization and impacts of the lattice structure in additive manufacturing. Mater. Today Proc..

[42] Tao W., Leu M.C. Design of lattice structure for additive manufacturing. Proceedings of the 2016 International Symposium on Flexible Automation (ISFA).

[43] Du Plessis A., Yadroitsava I., Yadroitsev I., Le Roux S., Blaine D. (2018). Numerical comparison of lattice unit cell designs for medical implants by additive manufacturing. Virtual Phys. Prototyp..

[44] Marschall D., Rippl H., Ehrhart F., Schagerl M. (2020). Boundary conformal design of laser sintered sandwich cores and simulation of graded lattice cells using a forward homogenization approach. Mater. Des..

[45] Teimouri M., Asgari M. (2020). Mechanical performance of additively manufactured uniform and graded porous structures based on topology-optimized unit cells. Proc. Inst. Mech. Eng. Part C J. Mech. Eng. Sci..

[46] Maskery I., Aboulkhair N., Aremu A., Tuck C., Ashcroft I. (2017). Compressive failure modes and energy absorption in additively manufactured double gyroid lattices. Addit. Manuf..

[47] Kumar A., Collini L., Daurel A., Jeng J.-Y. (2020). Design and additive manufacturing of closed cells from supportless lattice structure. Addit. Manuf..

[48] Yang L., Han C., Wu H., Hao L., Wei Q., Yan C., Shi Y. (2020). Insights into unit cell size effect on mechanical responses and energy absorption capability of titanium graded porous structures manufactured by laser powder bed fusion. J. Mech. Behav. Biomed. Mater..

[49] Cheng L., Liang X., Belski E., Wang X., Sietins J.M., Ludwick S., To A.C. (2018). Natural frequency optimization of variable-density additive manufactured lattice structure: Theory and experimental Validation. J. Manuf. Sci. Eng..

[50] Vantyghem G., De Corte W., Steeman M., Boel V. (2019). Density-based topology optimization for {3D}-printable building structures. Struct. Multidiscip. Optim..

[51] Plocher J., Panesar A. (2020). Effect of density and unit cell size grading on the stiffness and energy absorption of short fibre-reinforced functionally graded lattice structures. Addit. Manuf..

[52] Al-Ketan O., Lee D.-W., Rowshan R., Abu Al-Rub R.K. (2020). Functionally graded and multi-morphology sheet TPMS lattices: Design, manufacturing, and mechanical properties. J. Mech. Behav. Biomed. Mater..

[53] Singh G., Pandey P.M. (2019). Uniform and graded copper open cell ordered foams fabricated by rapid manufacturing: Surface morphology, mechanical properties and energy absorption capacity. Mater. Sci. Eng. A.

[54] Maskery I., Aremu A., Parry L., Wildman R., Tuck C., Ashcroft I. (2018). Effective design and simulation of surface-based lattice structures featuring volume fraction and cell type grading. Mater. Des..

[55] Al-Ketan O., Abu Al-Rub R.K. (2020). MSLattice: A free software for generating uniform and graded lattices based on triply periodic minimal surfaces. Mater. Des. Process. Commun..

[56] Dong G., Tang Y., Zhao Y.F. (2017). A Survey of modeling of lattice structures fabricated by additive manufacturing. J. Mech. Des..

[57] Montoya-Zapata D., Moreno A., Pareja-Corcho J., Posada J., Ruiz-Salguero O. (2019). Density-sensitive implicit functions using sub-voxel sampling in additive manufacturing. Metals.

[58] Chougrani L., Pernot J.-P., Véron P., Abed S. (2017). Lattice structure lightweight triangulation for additive manufacturing. Comput. Des..

[59] Xue D., Zhu Y., Guo X. (2020). Generation of smoothly-varying infill configurations from a continuous menu of cell patterns and the asymptotic analysis of its mechanical behaviour. Comput. Methods Appl. Mech. Eng..

[60] Autodesk NETFABB. https://www.autodesk.com/products/netfabb/.

[61] Altair. https://www.altair.com/additive-manufacturing/.

[62] Ansys Spaceclaim. https://www.ansys.com/products/3d-design/ansys-spaceclaim.

[63] Materialize 3-Matic. https://www.materialise.com/en/software/3-matic.

[64] ParaMatters. https://paramatters.com/.

[65] Garber T., Goldenberg J., Libai B., Muller E. (2004). Design and analysis of a mask projection micro- stereolithography system. Mark. Sci..

[66] Jacobson A., Panozzo D. A Simple C++ Geometry Processing Library. https://github.com/libigl/libigl.

[67] Fabri A., Pion S. (2009). CGAL—The computational geometry algorithms library: Demo paper. Proceedings of the 17th SIGSPATIAL International Conference on Advances in Geographic Information Systems.

[68] G-Truc Creation OpenGL Mathematics (GLM). https://glm.g-truc.net.

[69] Bernstein G. Cork Boolean Library. https://github.com/gilbo/cork.

[70] Guennebaud G., Jacob B. Eigen v3: Linear algebra library. http://eigen.tuxfamily.org.

[71] Winkle L. Van Tiny recursive descent expression parser, compiler, and evaluation engine for math expressions. https://codeplea.com/tinyexpr.

[72] Koc B., Lee Y.-S. (2002). Non-uniform offsetting and hollowing objects by using biarcs fitting for rapid prototyping processes. Comput. Ind..

[73] Rigid Cellular (2014). Plastics—Determination of Compression Properties.

[74] Garcia E.A., Ayranci C., Qureshi A.J. (2020). Material property-manufacturing process optimization for form 2 vat-photo polymerization 3D Printers. J. Manuf. Mater. Process..

[75] (2002). Plastics—Determination of Compressive Properties.

[76] (2011). Mechanical Testing of Metals—Ductility Testing—Compression Test for Porous and Cellular Metals.

[77] Mostafa K.G., Qureshi A.J. (2021). Dual Graded Lattice Structures Framework: STL Files.

